# Radical vulvectomy with a bilateral pudendal flap in the treatment of a vulvar cancer relapse

**DOI:** 10.1002/ccr3.770

**Published:** 2017-01-04

**Authors:** Begoña Díaz de la Noval

**Affiliations:** ^1^ Gynecology Oncology Unit FellowDepartment of Gynecology and ObstetricsLa Paz University Hospital‐IdiPAZPaseo de la Castellana 26128046MadridSpain

**Keywords:** General surgery, obstetrics and gynecology, oncology

## Abstract

One of the main risk factors for relapse in vulvar cancer after lymph‐node metastases is free surgical margins. In the case of a relapse, radical vulvectomy with perineal reconstruction is the first choice. Perineal reconstruction is usually indicated in relapse and unusual for a first surgery, except extensive damage 1, 2, 3, 4, 5.

## Clinical Image Description

**Figure 1:**
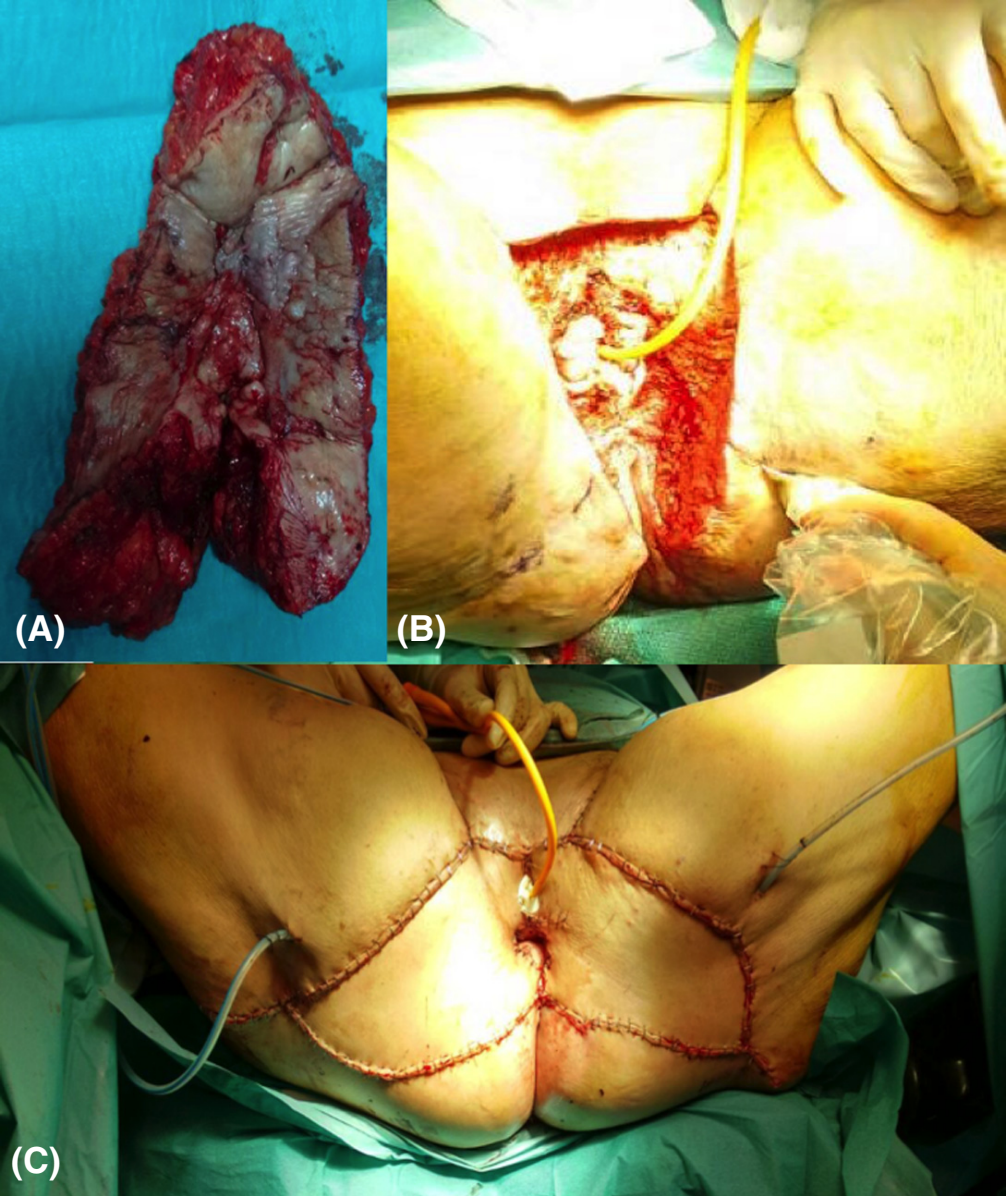
Radical vulvectomy with reconstruction by bilateral pudendal fasciocutaneous flap. (A) En block tumor resection. (B) Perineal area after exeresis and before reconstruction. (C) Final view after bilateral pudendal fasciocutaneous flap. We are used to placing two to four drainages from the perineal and flap area.

**Figure 2:**
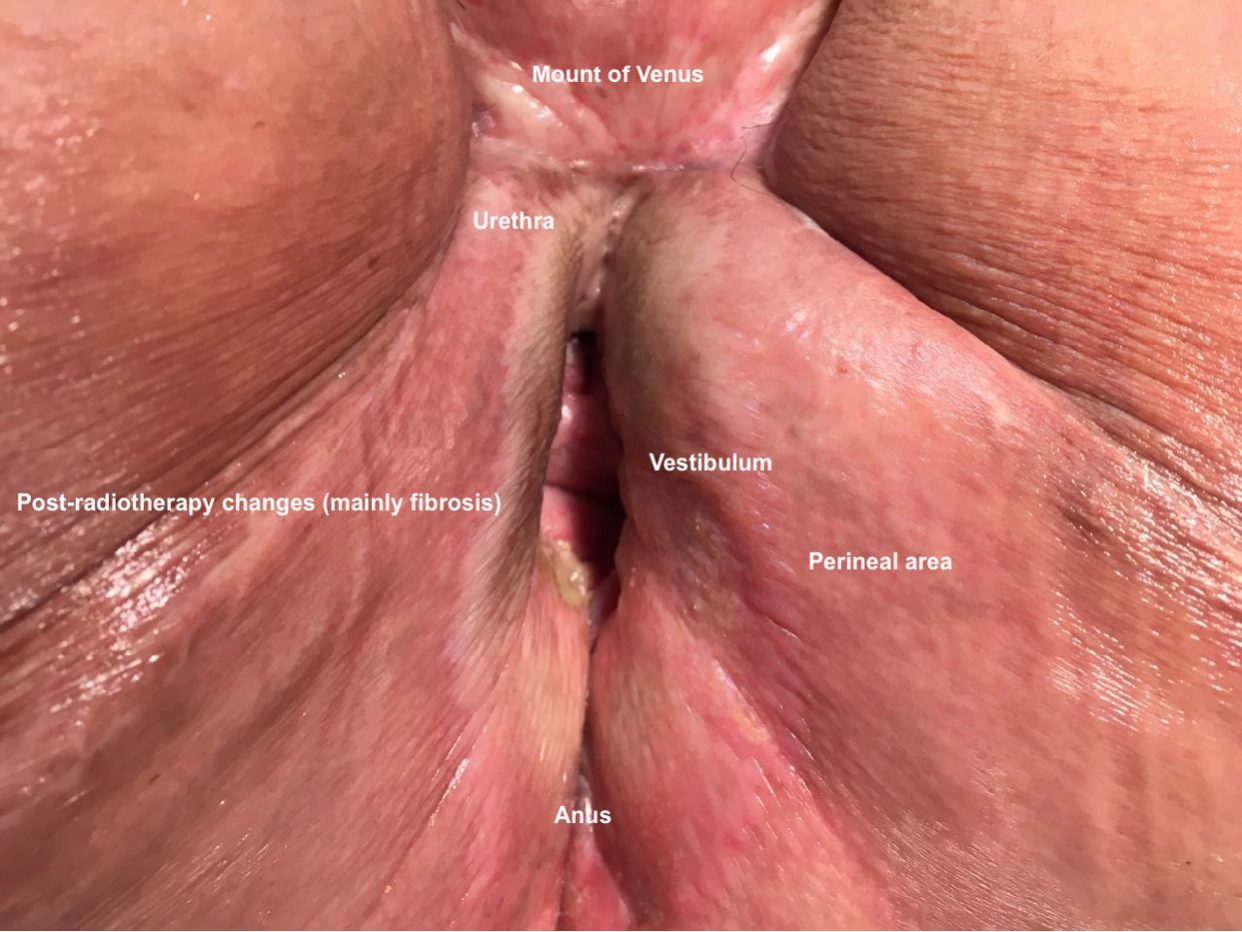
Follow‐up, perineal area a year after surgery. The photograph was taken a year later of surgery for relapse. A cicatricial vulva with changes secondary to radiotherapy. No signs of local relapse.

A 68‐year‐old woman was referred to our institution to assess for radiotherapy in a vulvar cancer relapse, two years after she was treated with radical vulvectomy and had no metastatic lymph node after performing the sentinel lymph‐node technique. She received adjuvant external‐beam radiotherapy because of a medial surgical margin <8 mm from tumor (surgical margin should be at least 20 mm). On gynecological examination, the vulvar region was erythematous, edematous, hard, and friable; with extensive tumor necrosis and damage that occupied the entire surface of the perineum and lower third of the vagina, there was no lymphadenopathy. The biopsy result was an invasive undifferentiated squamous carcinoma. Because the patient had received previous radiotherapy (during first‐line treatment at diagnosis) and recommended treatment of a central relapse according to international guidelines was an extended radical surgery with immediate reconstruction if requires, our patient performed a bilateral pudendal fasciocutaneous flap (Figure 1), and no adjuvant therapy was recommended (systemic therapy was not required). Postoperative healing was complicated because of prior radiotherapy, and perineal tissue was fibrotic and hypovascularized. Finally, she obtained a good aesthetic result with an acceptable quality of life (Figure 2), currently free of disease.

## Authorship

Begoña Díaz de la Noval performed the project development, manuscript writing/editing, manuscript review, literature search, and final approval of manuscript.

## Conflict of Interest

None declared.
